# Use of E-Cigarettes and Cigarettes During Late Pregnancy Among Adolescents

**DOI:** 10.1001/jamanetworkopen.2023.47407

**Published:** 2023-12-13

**Authors:** Xiaozhong Wen, Lufeiya Liu, Aye A. Moe, Isabelle K. Ormond, Chelsea C. Shuren, I’Yanna N. Scott, Jenny E. Ozga, Cassandra A. Stanton, Andrea L. Ruybal, Joy L. Hart, Maciej L. Goniewicz, Dara Lee, Comreen Vargees

**Affiliations:** 1 Division of Behavioral Medicine, Department of Pediatrics, Jacobs School of Medicine and Biomedical Sciences, State University of New York at Buffalo, Buffalo; 2 Department of Biostatistics, School of Public Health, University of Michigan, Ann Arbor; 3 Rochester Institute of Technology, Rochester, New York; 4 Behavioral Health and Health Policy, Westat, Rockville, Maryland; 5 Center for Tobacco Products, Food and Drug Administration, Silver Spring, Maryland; 6 Department of Communication, College of Arts and Sciences, University of Louisville, Louisville, Kentucky; 7 Department of Health Behavior, Roswell Park Comprehensive Cancer Center, Buffalo, New York; 8 New York Medical College, Valhalla

## Abstract

**Importance:**

Pregnant adolescents sometimes use cigarettes; however, little is known about e-cigarette use among pregnant adolescents, a population with increased health vulnerability.

**Objective:**

To examine yearly trends, sociodemographic and pregnancy-related determinants, and the association with small-for-gestational-age (SGA) birth of e-cigarette and/or cigarette use during late pregnancy among adolescents.

**Design, Setting, and Participants:**

This cohort study used existing data from the 2016-2021 Pregnancy Risk Assessment Monitoring System on 10 428 US adolescents aged 10 to 19 years who had a singleton birth with complete data on e-cigarette or cigarette use and SGA birth.

**Exposure:**

Adolescents reported e-cigarette and cigarette use during the last 3 months of pregnancy.

**Main Outcomes and Measures:**

SGA birth (birth weight below the 10th percentile for the same sex and gestational duration) was determined from birth certificates. Multivariable logistic regression was used to compare the odds of SGA birth across pregnant adolescents who exclusively used e-cigarettes, exclusively used cigarettes, used e-cigarettes and cigarettes, or did not use either.

**Results:**

Of the 10 428 pregnant adolescents, 72.7% were aged 18 or 19 years; 58.9% self-identified as White and 23.3% as Black; and 69.8% were non-Hispanic. The weighted prevalence of exclusive e-cigarette use during late pregnancy increased from 0.8% in 2016 to 4.1% in 2021, while the prevalence of exclusive cigarette use decreased from 9.2% in 2017 to 3.2% in 2021. The prevalence of dual use fluctuated, ranging from 0.6% to 1.6%. White pregnant adolescents were more likely than those who self-identified as another race and ethnicity to use e-cigarettes (2.7% vs 1.0% for American Indian or Alaska Native adolescents, 0.8% for Asian or other race adolescents, 0.6% for Black adolescents, and 0.7% for multiracial adolescents). Compared with those who did not use either product, adolescents who exclusively used e-cigarettes (16.8% vs 12.9%; confounder-adjusted odds ratio [AOR], 1.68 [95% CI, 0.89-3.18]) or who used cigarettes and e-cigarettes (17.6% vs 12.9%; AOR, 1.68 [95% CI, 0.79-3.53]) had no statistically significant difference in risk of SGA birth. However, adolescents who exclusively used cigarettes had a more than 2-fold higher risk of SGA birth (24.6% vs 12.9%; AOR, 2.51 [95% CI, 1.79-3.52]).

**Conclusions and Relevance:**

This cohort study suggests that pregnant adolescents increasingly used e-cigarettes, with the highest use among White adolescents. Results from this analysis found that, unlike cigarette use, e-cigarette use during late pregnancy was not statistically significantly associated with an increased risk of SGA birth among adolescents. Due to the uncertainty of this nonsignificant association, future research could benefit from a larger sample size.

## Key Points

**Question:**

What are the trends, determinants, and association with small-for-gestational-age (SGA) birth of e-cigarette use among pregnant adolescents?

**Findings:**

This cohort study using 2016-2021 data on 10 428 adolescents from the Pregnancy Risk Assessment Monitoring System in the US found that pregnant adolescents have increasingly used e-cigarettes, with the highest use among White adolescents. Adolescent use of cigarettes during pregnancy was a risk factor for SGA birth; however, adolescent use of e-cigarettes or dual use of e-cigarettes and cigarettes was not associated with SGA birth.

**Meaning:**

This study suggests that e-cigarette use during late pregnancy among adolescents was not statistically significantly associated with a high risk of SGA birth.

## Teaser

This cohort study uses data from the Pregnancy Risk Assessment Monitoring System to examine yearly trends, sociodemographic and pregnancy-related determinants, and the association with small-for-gestational-age birth of e-cigarette and/or cigarette use during late pregnancy among adolescents.

## Introduction

Cigarette smoking sometimes occurs among pregnant adolescents; 4.5% of people who gave birth in the US in 2021 smoked cigarettes during pregnancy, with the highest prevalence among young adults aged 20 to 24 years (5.8%) or 25 to 29 years (5.1%), followed by adolescents aged 15 to 19 years (4.3%).^1^ E-cigarettes are another major tobacco or nicotine product used by US adolescents.^2,3^ According to the 2022 National Youth Tobacco Survey, 3.3% of middle school students and 14.1% of high school students used e-cigarettes that year,^2^ although it is unclear how common e-cigarette use is among pregnant adolescents.

Nicotine, carbon monoxide, and other chemicals in combustible cigarettes may contribute to the maternal smoking–related risk to the fetus.^4,5^ E-cigarettes are noncombustible and do not generate some of the toxic chemicals present in tobacco smoke, including carbon monoxide.^6^ For this reason, some pregnant people who smoke cigarettes before pregnancy use e-cigarettes as a smoking cessation aid to reduce nicotine intake and/or to reduce harm to the fetus.^7^ However, average nicotine delivery from e-cigarettes is comparable to that of combustible cigarettes.^8^ In addition, despite being 1 to 2 orders of magnitude lower than levels from combustible cigarette products, trace levels of certain toxicants, including formaldehyde, acetaldehyde, nickel, and lead, are present in e-cigarettes.^9^ The presence of those toxicants in emissions from e-cigarettes may explain, at least in part, why e-cigarette use during pregnancy is associated with adverse birth outcomes, such as low birth weight, preterm birth, and small-for-gestational-age (SGA) birth among people who gave birth, including adolescents and adults^10^ and adults aged 18 years or older.^11^

SGA birth, defined as birth weight below the 10th percentile for the same sex and gestational duration, is a public health concern in the US and globally.^12,13,14,15^ Between 2012 and 2016, the prevalence of SGA birth was as high as 12.1% in the US.^16^ SGA infants are at an increased risk for adverse health outcomes, including postnatal growth failure,^17^ neurodevelopmental impairment,^18^ short stature,^19^ and type 2 diabetes.^20^

One study showed that infants of adolescents who smoked were 3.1 times more likely to be SGA compared with infants of adolescents who did not smoke cigarettes.^21^ Compared with adults who have given birth, adolescents may be more vulnerable to smoking-related health risks due to the biological and psychosocial factors pertaining to adolescent pregnancies. One prior study showed that infants of adolescents (aged 12-18 years) who smoked during pregnancy had a greater reduction in birth weight than infants of adults who smoked during pregnancy (−202 g vs −158 g per pack per day).^22^ Despite existing findings on the adverse health effects of cigarette smoking among pregnant adolescents, little is known about e-cigarette use among this population with increased health vulnerability.

Therefore, using a large US national pregnancy monitoring system, we aimed to fill the aforementioned research gaps by examining exclusive e-cigarette use, exclusive cigarette use, and dual use of e-cigarettes and cigarettes during pregnancy among adolescents. We focused on yearly trends in use from 2016 to 2021, sociodemographic and pregnancy-related determinants, and a potential health outcome of pregnancy (SGA birth).

## Methods

### Population and Sample

In this cohort study, we conducted a secondary data analysis using Phase 8 of the US Pregnancy Risk Assessment Monitoring System (PRAMS; 2016-2021). The PRAMS is an ongoing state-level, population-based surveillance system first administered in 1987.^23^ It applies a mixed-mode approach of birth certificates, mailed surveys, and telephone surveys to collect information on maternal behaviors, attitudes, and experiences before, during, and shortly after (2-6 months) pregnancy. This information is all collected retrospectively after live birth and documentation of SGA status is available. Approximately 83% of all US births are covered by the PRAMS, including 47 states, the District of Columbia, New York City, Puerto Rico, and the Great Plains Tribal Chairman’s Health Board.^23^ The deidentified PRAMS data were provided by the Centers for Disease Control and Prevention. This secondary data analysis was approved as non–human participants research by the University at Buffalo institutional review board and did not require informed consent from study participants. This report followed the Strengthening the Reporting of Observational Studies in Epidemiology (STROBE) reporting guideline.

The total sample size of the Phase 8 PRAMS from 2016 to 2021 was 242 573 (eFigure in Supplement 1). First, given the significantly distinct developmental and health outcomes associated with multiple births, we excluded these data and focused solely on singleton births. Then we applied the age criterion for adolescence (10-19 years) defined by the World Health Organization.^24,25^ Among these adolescents, we included only those with complete data on e-cigarette and cigarette use during the last 3 months of pregnancy (late pregnancy). Furthermore, we restricted the analytic sample to adolescents who had complete data on SGA birth.

### Exposure Measures

For e-cigarette use during late pregnancy, participants were asked the following question, “During the last 3 months of your pregnancy, on average, how often did you use e-cigarettes or other electronic nicotine products?” with response options of (1) more than once a day, (2) once a day, (3) 2 to 6 days a week, (4) 1 day a week or less, and (5) did not use e-cigarettes or other electronic nicotine products then. The questionnaire included a note that defined e-cigarettes as follows: “E-cigarettes (electronic cigarettes) and other electronic nicotine vaping products (such as vape pens, e-hookahs, hookah pens, e-cigars, e-pipes) are battery-powered devices that use nicotine liquid rather than tobacco leaves, and produce vapor instead of smoke.” We dichotomized responses into e-cigarette use (options 1, 2, 3, or 4) or nonuse (option 5) during late pregnancy to ensure sufficient statistical power for further analyses on determinants of e-cigarette use as well as its association with SGA birth. For cigarette use during late pregnancy, participants were asked the following question, “In the last 3 months of your pregnancy, how many cigarettes did you smoke on an average day?” with response options of (1) 41 cigarettes or more, (2) 21 to 40 cigarettes, (3) 11 to 20 cigarettes, (4) 6 to 10 cigarettes, (5) 1 to 5 cigarettes, (6) less than 1 cigarette, and (7) did not smoke then. Similar to e-cigarette use, we dichotomized responses into cigarette use (options 1-6) or nonuse (option 7) during late pregnancy for further analyses.

Based on e-cigarette and cigarette use status during late pregnancy, we further categorized participants into 4 mutually exclusive groups: adolescents who did not use either e-cigarettes or cigarettes, those who exclusively used e-cigarettes, those who exclusively used cigarettes, and those who used both e-cigarettes and cigarettes (dual use).

### Outcome Measures

Using data from birth certificates, the percentiles of birth weight by sex and gestational duration were calculated using the natality files for singleton births from the National Center for Health Statistics.^26^ We defined SGA birth as birth weight below the 10th percentile for the same sex and gestational duration, according to the cutoff point proposed by the World Health Organization.^27,28^ This definition of SGA has been applied in previous research using the PRAMS data.^29^

### Potential Determinants of E-Cigarette Use and Confounders

Based on the relevant literature,^30,31,32,33^ we considered the following sociodemographic and pregnancy-related characteristics as potential determinants of e-cigarette use during late pregnancy: age (10-17 years and 18 or 19 years), race (American Indian or Alaska Native, Asian or other race [no further information was provided for the category of “other race”], Black, multiracial, White), ethnicity (Hispanic or non-Hispanic), marital status (unmarried or married), type of health insurance (Medicaid, private insurance, self-pay, or other), prepregnancy hypertension (yes or no), prepregnancy diabetes (yes or no), prepregnancy body mass index (BMI; calculated as weight in kilograms divided by height in meters squared), and the child’s birth year (2016-2021). In addition, these characteristics were considered as potential confounders in the associations between e-cigarette use and risk of SGA birth, given that they also have been reported to be associated with SGA in previous research.^34,35,36,37^

Race and ethnicity were included due to known disparities in the prevalence of nicotine product use^30,33^ and adverse birth outcomes.^37,38^ Race and ethnicity were obtained from birth certificates. Due to small sample sizes, we combined the American Indian and Alaska Native categories into a single category. Similarly, the Asian and other race categories were combined into a separate single category. Additional information on selection of potential determinants can be found in the eMethods in Supplement 1.

### Statistical Analysis

Information on the descriptive analysis can be found in the eMethods in Supplement 1. We used logistic regression models to examine whether the prevalence of e-cigarette and/or cigarette use significantly varied between 2016 and 2021. We used χ^2^ tests for categorical variables (eg, race) and a linear regression model for BMI, a continuous variable, to identify significant sociodemographic and pregnancy-related determinants of e-cigarette and/or cigarette use. Similarly, χ^2^ tests were used to compare the risk of SGA birth by categorical sociodemographic and pregnancy-related characteristics. For prepregnancy BMI (continuous), we compared its mean between SGA birth and non-SGA births using a linear regression model. We also used χ^2^ tests to compare the risk of SGA birth across the 4 tobacco use groups. Then, we fitted multivariable binary logistic regression models to estimate the associations between e-cigarette and/or cigarette use with the risk of SGA birth. We calculated crude odds ratios (ORs) and confounder-adjusted ORs (AORs) and 95% CIs of SGA birth for adolescents who exclusively used e-cigarettes, exclusively used cigarettes, or used both cigarettes and e-cigarettes compared with adolescents who did not use either product (reference group). The confounders included in the adjusted model were maternal age, race, ethnicity, marital status, health insurance, prepregnancy BMI, prepregnancy diabetes, prepregnancy hypertension, and the child’s birth year, based on the literature on factors associated with nicotine product use and risk of SGA birth.^30,31,32,33,34,35,36,37^

To study the possible effect modification of prepregnancy BMI or the child’s birth year in the association of e-cigarette and/or cigarette use with the risk of SGA birth, we added interaction terms between prepregnancy BMI or the child’s birth year and e-cigarette or cigarette use to subsequent regression models. We focused on these 2 potential effect modifiers because previous research showed that the magnitude of the association between cigarette smoking during pregnancy and risk of SGA birth among pregnant adolescents who were underweight or normal weight was more striking than among those who were overweight or obese^39^ and because e-cigarette devices have substantially changed through the recent years and thus the potential association of maternal use of e-cigarette products during pregnancy with fetal growth may have been changing with the child’s birth year as well. In a sensitivity analysis to evaluate the robustness of our analytic results, we additionally excluded 36 adolescents (0.4%) who initiated the use of e-cigarettes (n = 25) or cigarettes (n = 11) during pregnancy so that the results could be interpreted as continuous use during pregnancy.

All data analyses were performed using SAS, version 9.4 (SAS Institute Inc). We defined statistical significance as 2-sided *P* < .05. Sampling weights were used in the statistical analysis to reduce potential selection bias due to nonrandom sampling, noncoverage, and nonresponse.^23^

## Results

### Sample Characteristics

In the original PRAMS sample of 242 573 births, 229 176 were singletons (eFigure in Supplement 1). Among these singletons, 10 746 were birthed by adolescents aged 10 to 19 years. Among these adolescents, 10 451 had complete data on e-cigarette and cigarette use during late pregnancy and 10 428 of these had complete data on SGA birth (the final analytic sample). Table 1 shows the sociodemographic and pregnancy-related characteristics of the 10 428 pregnant adolescents in the analytic sample. Among them, 27.3% were aged 10 to 17 years and 72.7% were aged 18 or 19 years, 58.9% self-identified as White and 23.3% as Black, 69.8% were non-Hispanic, 91.6% were unmarried, and 78.5% had Medicaid insurance. The children’s birth years were evenly distributed from 2016 to 2021, with approximately 17% of the sample occurring each year.

**Table 1.** Characteristics of Adolescents With Live Singleton Births

Characteristic^a^	No. (weighted %)				
	Total sample (N = 10 428)	Nonusers (n = 9432)	EC users (n = 152)	CC users (n = 719)	Dual users (n = 125)
Age, y					
10-17	2778 (27.3)	2599 (28.0)	35 (22.1)	120 (20.1)	24 (16.8)
18 or 19	7650 (72.7)	6833 (72.0)	117 (77.9)	599 (79.9)	101 (83.2)
Race					
American Indian or Alaska Native	956 (2.0)	826 (2.0)	16 (1.2)	107 (2.0)	7 (4.2)
Asian or other race^b^	961 (10.7)	937 (11.6)	4 (4.4)	18 (1.9)	2 (0.3)
Black	2702 (23.3)	2592 (25.2)	13 (7.0)	88 (6.5)	9 (5.1)
Multiracial	887 (5.1)	769 (5.1)	15 (2.0)	75 (5.0)	28 (9.6)
White	4836 (58.9)	4224 (56.1)	104 (85.5)	429 (84.7)	79 (80.9)
Ethnicity					
Hispanic	3115 (30.2)	3018 (32.4)	25 (17.5)	61 (8.6)	11 (4.4)
Non-Hispanic	7271 (69.8)	6378 (67.6)	126 (82.5)	653 (91.4)	114 (95.6)
Marital status					
Unmarried	9578 (91.6)	8649 (91.4)	140 (93.5)	671 (92.2)	118 (98.3)
Married	841 (8.4)	775 (8.6)	12 (6.5)	47 (7.8)	7 (1.7)
Type of health insurance					
Medicaid	8230 (78.5)	7435 (78.1)	112 (76.0)	576 (83.2)	107 (89.8)
Private, self-pay, or other	2111 (21.5)	1924 (21.9)	39 (24.0)	131 (16.8)	17 (10.2)
Prepregnancy hypertension	1143 (9.1)	1037 (9.2)	19 (8.6)	75 (8.7)	12 (5.1)
Prepregnancy diabetes	243 (2.0)	225 (2.1)	0 (0.0)	15 (1.4)	3 (0.3)
Prepregnancy BMI, mean (SE)	25.0 (0.1)	25.0 (0.1)	25.1 (0.6)	24.5 (0.4)	24.3 (0.2)
Child’s birth year					
2016	1693 (19.0)	1523 (19.0)	13 (8.7)	136 (20.5)	21 (20.0)
2017	1818 (16.1)	1620 (15.7)	19 (10.9)	156 (21.9)	23 (22.1)
2018	2028 (17.1)	1829 (17.0)	15 (10.5)	161 (21.4)	23 (13.6)
2019	1783 (18.2)	1623 (18.2)	25 (20.1)	115 (17.8)	20 (24.4)
2020	1673 (16.2)	1520 (16.5)	33 (19.4)	100 (12.0)	20 (7.6)
2021	1433 (13.4)	1317 (13.6)	47 (30.3)	51 (6.4)	18 (12.3)
Frequency of EC use during the last 3 mo of pregnancy					
More than once a day	90 (1.0)	0	47 (29.9)	0	43 (38.6)
Once a day	34 (0.5)	0	26 (24.9)	0	8 (1.3)
2-6 d/wk	39 (0.3)	0	20 (10.2)	0	19 (7.2)
≤1 d/wk	114 (1.3)	0	59 (34.9)	0	55 (52.8)
Nonuse of EC	10151 (97.0)	9432 (100.0)	0	719 (100.0)	0
Daily frequency of CC use during the last 3 mo of pregnancy					
≥41 Cigarettes	4 (0.01)	0	0	3 (0.1)	1 (0.4)
21-40 Cigarettes	12 (0.1)	0	0	6 (0.9)	6 (3.5)
11-20 Cigarettes	59 (0.5)	0	0	49 (6.5)	10 (1.8)
6-10 Cigarettes	149 (1.7)	0	0	129 (22.1)	20 (19.0)
1-5 Cigarettes	432 (3.9)	0	0	372 (49.2)	60 (53.5)
<1 Cigarette	188 (1.7)	0	0	160 (21.2)	28 (21.7)
Nonuse of CC	9584 (92.1)	9432 (100.0)	152 (100.0)	0	0

Abbreviations: BMI, body mass index (calculated as weight in kilograms divided by height in meters squared); CC, combustible cigarette; EC, e-cigarette.

^a^ Sum of categories may not be equal to the total due to missing data.

^b^ No additional information was provided about the races included in the “other race” category.

### Prevalence of E-Cigarette and/or Cigarette Use During Late Pregnancy

As shown in Table 2, 1.5% of the total sample exclusively used e-cigarettes, 7.3% exclusively used cigarettes, 1.2% used cigarettes and e-cigarettes, and the remaining 90.1% did not use either product during late pregnancy. Among those who reported exclusive e-cigarette use, 34.9% used e-cigarettes 1 day per week or less and 29.9% used e-cigarettes more than once a day (Table 1). Among those who reported exclusive cigarette use, 49.2% used 1 to 5 cigarettes per day and 22.1% used 6 to 10 cigarettes per day.

**Table 2.** Determinants of EC and/or CC Use During Late Pregnancy Among Adolescents, and Their Risk of Small-for-Gestational-Age Birth

Determinants	EC or CC use during late pregnancy					Small-for-gestational-age birth	
	Weighted %				*P* value^a^	Weighted %	*P* value^a^
	Nonuse	EC use	CC use	Dual use			
Total sample	90.1	1.5	7.3	1.2	NA	13.9	NA
Age, y							
10-17	92.8	1.5	5.0	0.7	.01	14.3	.54
18 or 19	89.3	2.0	7.4	1.3		13.6	
Race							
American Indian or Alaska Native	90.0	1.0	6.5	2.4	<.001	10.0	.33
Asian or other race^b^	98.0	0.8	1.2	0.0		15.2	
Black	97.3	0.6	1.9	0.3		13.1	
Multiracial	90.3	0.7	6.7	2.2		14.3	
White	85.9	2.7	9.8	1.6		13.7	
Ethnicity							
Hispanic	96.8	1.1	1.9	0.2	<.001	14.8	.22
Non-Hispanic	87.4	2.2	8.9	1.6		13.3	
Marital status							
Unmarried	90.1	1.9	6.8	1.3	<.001	14.0	.04
Married	92.1	1.4	6.3	0.2		10.7	
Type of health insurance							
Medicaid	89.8	1.8	7.1	1.4	.01	13.5	.33
Private, self-pay, or other	92.2	2.1	5.2	0.6		14.9	
Prepregnancy hypertension							
Yes	91.1	1.7	6.5	0.7	.46	16.1	.19
No	90.2	1.8	6.8	1.2		13.6	
Prepregnancy diabetes							
Yes	95.2	0.0	4.7	0.2	Inestimable	11.8	.56
No	90.1	1.9	6.8	1.2		13.8	
Prepregnancy BMI, mean (SE)	25.0 (0.1)	25.1 (0.6)	24.5 (0.4)	24.3 (0.2)	.53	23.8 (0.2)^c^	<.001
Child’s birth year							
2016	90.6	0.8	7.3	1.2	<.001	14.5	.38
2017	88.0	1.2	9.2	1.6		15.4	
2018	89.5	1.1	8.4	0.9		14.6	
2019	89.8	2.0	6.6	1.6		11.9	
2020	92.2	2.2	5.0	0.6		13.2	
2021	91.6	4.1	3.2	1.1		12.9	

Abbreviations: BMI, body mass index (calculated as weight in kilograms divided by height in meters squared); CC, combustible cigarette; EC, e-cigarette; NA, not applicable.

^a^ Calculated from χ^2^ tests for categorical determinants or from a linear regression model for the continuous determinant (prepregnancy BMI). Statistical significance was defined as a 2-sided *P* < .05.

^b^ No additional information was provided about the races included in the “other race” category.

^c^ The mean (SE) prepregnancy BMI for adolescents with non-SGA birth was 25.2 (0.1).

In 2021, the prevalence of use during late pregnancy among adolescents was highest for exclusive e-cigarette use (4.1%), followed by exclusive cigarette use (3.2%), and lowest for dual cigarette and e-cigarette use (1.1%) (Figure). The prevalence of exclusive e-cigarette use increased significantly from 0.8% in 2016 to 4.1% in 2021 (*P* = .001 for the trend test). Statistically significant pairwise comparisons for e-cigarette use included 2021 vs 2016, 2021 vs 2017, and 2021 vs 2018. In contrast, the prevalence of exclusive cigarette use gradually decreased from 9.2% in 2017 to 3.2% in 2021 (*P* < .001 for the trend test). Statistically significant pairwise comparisons for cigarette use included 2020 vs 2017, 2020 vs 2018, 2021 vs 2016, 2021 vs 2017, 2021 vs 2018, and 2021 vs 2019. The prevalence of dual use of cigarettes and e-cigarettes varied across the years, with a range from 0.6% to 1.6% (*P* = .38 for the trend test). There were no significant pairwise comparisons for dual use between any 2 years.

**Figure.**
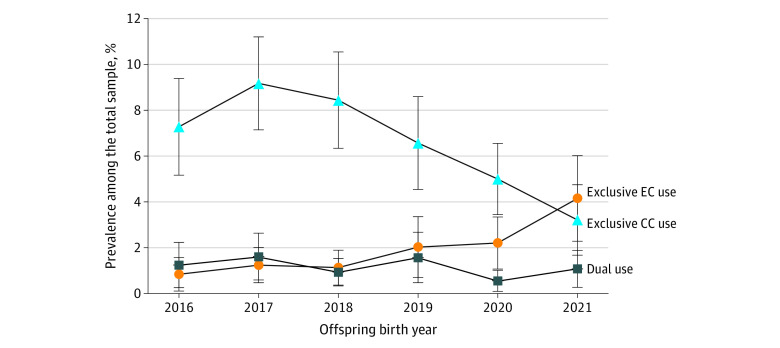
Prevalence of E-Cigarette (EC) or Combustible Cigarette (CC) Use During Late Pregnancy Among Adolescents by Birth Year, 2016-2021 The analytic sample size was 1693 in 2016, 1818 in 2017, 2028 in 2018, 1783 in 2019, 1673 in 2020, and 1433 in 2021. The trend test *P* values were *P* < .001 for exclusive e-cigarette use from 2016 to 2021, *P* < .001 for exclusive cigarette use from 2017 to 2021, and *P* = .38 for dual use from 2016 to 2021. Error bars indicate 95% CIs.

### Determinants of E-Cigarette and/or Cigarette Use During Late Pregnancy

Adolescents aged 18 or 19 years had a higher prevalence of exclusive e-cigarette use (2.0% vs 1.5%), exclusive cigarette use (7.4% vs 5.0%), and dual use (1.3% vs 0.7%) compared with those aged 10 to 17 years (Table 2). White adolescents had the highest prevalence of exclusive e-cigarette use (2.7%) and exclusive cigarette use (9.8%), whereas Black adolescents had the lowest prevalence of exclusive e-cigarette use (0.6%) and Asian or other race adolescents had the lowest prevalence of exclusive cigarette use (1.2%). Compared with Hispanic adolescents, non-Hispanic adolescents had a higher prevalence of exclusive e-cigarette use (2.2% vs 1.1%), exclusive cigarette use (8.9% vs 1.9%), and dual use (1.6% vs 0.2%). Compared with unmarried adolescents, married adolescents were less likely to exclusively use e-cigarettes (1.4% vs 1.9%) or cigarettes (6.3% vs 6.8%). Adolescents with Medicaid insurance were more likely than adolescents with other health insurance to exclusively use cigarettes (7.1% vs 5.2%) and use both e-cigarettes and cigarettes (1.4% vs 0.6%).

### Associations Between E-Cigarette and/or Cigarette Use During Late Pregnancy and SGA

In the total sample, 13.9% of children were born SGA (Table 2). Being unmarried (14.0% vs 10.7% being married) and a lower prepregnancy BMI were significant risk factors for SGA. Compared with adolescents who did not use either product, those who exclusively used e-cigarettes appeared to have no significantly different odds of SGA birth (16.8% vs 12.9%; crude OR, 1.37 [95% CI, 0.73-2.56]; AOR, 1.68 [95% CI, 0.89-3.18]) (Table 3). Similarly, those who used both e-cigarettes and cigarettes appeared to have no significant difference in the odds of SGA birth from those who did not use either product (17.6% vs 12.9%; crude OR, 1.45 [95% CI, 0.71-2.96]; AOR, 1.68 [95% CI, 0.79-3.53]). However, adolescents who exclusively used cigarettes had a more than 2-fold higher odds of SGA birth than those who did not use either product (24.6% vs 12.9%; crude OR, 2.21 [95% CI, 1.61-3.04]). After adjustment for confounders, exclusive cigarette use remained a risk factor for SGA birth (AOR, 2.51 [95% CI, 1.79-3.52]).

**Table 3.** Crude and Adjusted Associations Between EC and/or CC Use During Late Pregnancy and Small-for-Gestational-Age Birth Among Adolescents

Maternal CC or EC use during late pregnancy	Sample size, No.	Small-for-gestational-age birth				
		Weighted %	Crude OR (95% CI)	*P* value	Adjusted OR (95% CI)^a^	Adjusted *P* value
Nonuser	9432	12.9	1 [Reference]	NA	1 [Reference]	NA
EC user (vs nonuser)	152	16.8	1.37 (0.73-2.56)	.33	1.68 (0.89-3.18)	.11
CC user (vs nonuser)	719	24.6	2.21 (1.61-3.04)	<.001	2.51 (1.79-3.52)	<.001
Dual user (vs nonuser)	125	17.6	1.45 (0.71-2.96)	.31	1.68 (0.79-3.53)	.18
EC user	152	16.8	1 [Reference]	NA	1 [Reference]	NA
CC user (vs EC user)	719	24.6	1.62 (0.81-3.23)	.17	1.50 (0.74-3.02)	.26
Dual user (vs EC user)	125	17.6	1.06 (0.41-2.71)	.91	1.00 (0.38-2.62)	≥.99
CC user	719	24.6	1 [Reference]	NA	1 [Reference]	NA
Dual user (vs CC user)	125	17.6	0.65 (0.30-1.41)	.28	0.67 (0.30-1.49)	.32

Abbreviations: CC, combustible cigarette; EC, e-cigarette; NA, not applicable; OR, odds ratio.

^a^ Adjusted for maternal age, race, ethnicity, marital status, health insurance, prepregnancy body mass index, prepregnancy diabetes, prepregnancy hypertension, and the child’s birth year.

Prepregnancy BMI was inversely associated with the risk of SGA birth, with a higher BMI associated with a lower risk of SGA birth (AOR, 0.96 [95% CI 0.94-0.98] per kg/m^2^ increment) (Table 4). However, prepregnancy BMI did not modify the associations between e-cigarette and/or cigarette use and SGA. Similarly, the child’s birth year was not a significant effect modifier. Finally, results did not change meaningfully in our sensitivity analysis by excluding the 36 adolescents who initiated the use of e-cigarettes or cigarettes during pregnancy.

**Table 4.** Interactions Between Prepregnancy BMI or Child’s Birth Year and EC and/or CC Use During Late Pregnancy in the Risk of Small-for-Gestational-Age Birth Among Adolescents

Maternal EC or CC use during late pregnancy	Small-for-gestational-age brith			
	Crude OR (95% CI)	*P* value	Adjusted OR (95% CI)^a^	Adjusted *P* value
Interaction with prepregnancy BMI				
EC user (vs nonuser)	1.49 (0.79-2.81)	.22	1.69 (0.90-3.18)	.11
CC user (vs nonuser)	2.13 (1.53-2.97)	<.001	2.39 (1.69-3.39)	<.001
Dual user (vs nonuser)	1.56 (0.76-3.19)	.23	1.77 (0.86-3.66)	.12
Prepregnancy BMI^b^	0.96 (0.94-0.98)	<.001	0.96 (0.94-0.98)	<.001
EC user × prepregnancy BMI	1.05 (0.91-1.22)	.52	1.05 (0.90-1.21)	.54
CC user × prepregnancy BMI	0.97 (0.92-1.03)	.36	0.97 (0.91-1.03)	.33
Dual user × prepregnancy BMI	1.08 (1.00-1.16)	.06	1.08 (1.00-1.16)	.06
Interaction with the child’s birth year				
EC user (vs nonuser)	1.60 (0.46-5.59)	.47	1.65 (0.46-5.95)	.45
CC user (vs nonuser)	2.25 (1.29-3.91)	.004	2.46 (1.39-4.38)	.002
Dual user (vs nonuser)	1.50 (0.44-5.17)	.52	1.70 (0.47-6.11)	.42
The child’s birth year	0.97 (0.91-1.03)	.28	0.96 (0.91-1.03)	.25
EC user × the child’s birth year	0.96 (0.66-1.40)	.83	1.01 (0.69-1.47)	.97
CC user × the child’s birth year	0.99 (0.79-1.23)	.90	1.01 (0.80-1.27)	.93
Dual user × the child’s birth year	0.98 (0.65-1.47)	.91	0.99 (0.65-1.52)	.98

Abbreviations: BMI, body mass index (calculated as weight in kilograms divided by height in meters squared); CC, combustible cigarette; EC, e-cigarette; OR, odds ratio.

^a^ Adjusted for maternal age, race, ethnicity, marital status, health insurance, prepregnancy diabetes, prepregnancy hypertension, and the child’s birth year.

^b^ Prepregnancy BMI was centered at 25.0.

## Discussion

We found that the prevalence of e-cigarette use among US pregnant adolescents increased steadily from 2016 to 2021. This trend paralleled the increasing prevalence of e-cigarette use among all adolescents across similar years.^40,41^ Our finding that White adolescents were more likely than other racial groups to use e-cigarettes and cigarettes during late pregnancy was consistent with previous research in the general adolescent population.^42^ Another risk factor for cigarette use and dual use in our study sample was having Medicaid insurance (an indicator of low household income). This finding adds to the existing evidence on the high prevalence of smoking among adolescents with low socioeconomic status.^43^ In addition, we found that married adolescents were less likely than unmarried adolescents to exclusively use cigarettes or use both cigarettes and e-cigarettes, which was supported by the literature showing that being married was a protective factor against cigarette use and dual use during pregnancy among adults.^11^

We found that the risk of SGA birth was more than 2-fold higher among adolescents who exclusively used cigarettes during late pregnancy than those who did not. This finding was consistent with previous research showing that maternal cigarette use during pregnancy was a risk factor for SGA birth.^44^ Nicotine, carbon monoxide, and other harmful chemicals in cigarettes may contribute to this association.^4^ For instance, carbon monoxide, which is present in cigarettes but not in most e-cigarettes, can cause fetal hypoxia and thus lead to fetal growth restriction.^5^

A novel finding from our analysis was the statistically nonsignificant association between adolescent e-cigarette use during late pregnancy and the risk of SGA birth. This finding might be associated with the fact that e-cigarettes contain little carbon monoxide and potentially lower nicotine concentrations compared with cigarettes. Cigarettes produce 0.8 to 1.7 mg of carbon monoxide and 81.5 to 187.7 μg of nicotine per puff.^45,46,47,48^ However, e-cigarettes usually do not produce carbon monoxide above the limit of detection,^49^ and the estimated amount of nicotine is 0 to 52 mg/mL.^50^ Still, previous studies indicated that e-cigarette use during pregnancy may be a risk factor for SGA birth among adults (aged ≥18 years)^51^ and among adolescents and adults.^29^ For example, Cardenas et al^51^ found that adult mothers who used e-cigarettes during pregnancy had a several times higher risk of SGA birth than mothers who did not use e-cigarettes. The reasons for the seeming inconsistency with our observed nonsignificant association among adolescents are unknown, but there are several plausible explanations. First, adolescents may have a shorter duration and a lower cumulative amount of e-cigarette use than older adults. It has been reported that most US adolescents begin using e-cigarettes in middle school and high school,^52,53,54^ so it is possible that the adolescents in our sample had not been using e-cigarettes for very long. As a result, their exposure to e-cigarettes might influence them and their offspring less, compared with adults. Second, our statistical power might have been insufficient to detect significant associations due to the relatively small number of adolescents in the PRAMS sample who exclusively used e-cigarettes or used both e-cigarettes and cigarettes.

### Limitations

This study has some limitations. First, self-reported e-cigarette and cigarette use measures were subject to recall bias, and the prevalence might have gone underreported due to social stigma, especially given that respondents completed the surveys retrospectively during the 2- to 6-month postpartum period. Second, the sample sizes for adolescents who used e-cigarettes (n = 152 [1.5%, weighted]) or both e-cigarettes and cigarettes (n = 125 [1.2%, weighted]) during pregnancy were relatively small, which might widen the 95% CIs in our estimated risk of SGA birth among them; therefore, caution is needed to interpret those results. Third, information on e-cigarette and cigarette use during the first and second trimesters of pregnancy was not available in the PRAMS; thus, we could not examine the potential association of the timing of exposure during pregnancy with SGA births.^55,56^ This limitation also did not allow us to distinguish individuals who used e-cigarettes and/or cigarettes throughout pregnancy from individuals who used these products only during the third trimester. Fourth, there was a lack of information on secondhand smoke exposure during pregnancy, which also could have a negative association with fetal growth and might have been an unmeasured confounder.^57^ Fifth, information on specific e-cigarette devices used by the participants was not available in the PRAMS. Given the changes in the tobacco product market, it could be difficult to draw conclusions on specific products over time. Sixth, we did not control for use of other substances, such as cannabis, due to a large amount of missing data. Thus, we could not distinguish whether individuals used electronic delivery devices to vape nicotine, cannabis, or both substances. Use of all these products can occur among adolescents^58^ and potentially have different associations with fetal growth.^59^ This limitation is particularly important in interpreting the results for individuals who use both nicotine and cannabis. Our estimated OR for cigarette use might be overestimated, given the reported associations of cannabis use with SGA and low birth weight.^60,61^ Seventh, we dichotomized e-cigarette and cigarette use (use vs nonuse) due to limited sample sizes of the original frequency categories. This simplified approach created a heterogeneous use group with considerable variability in use frequency and did not allow us to examine the potential dose-response association with risk of SGA birth. Eighth, maternal diet quality could have a substantial association with fetal growth,^62,63^ but it was not considered in our analysis due to lack of information.

## Conclusions

In this cohort study of US pregnant adolescents, there was an increase in e-cigarette use and a decrease in cigarette use during late pregnancy from 2016 to 2021. In this population with a potentially higher risk of SGA birth, exclusive cigarette use was a risk factor for SGA birth. Exclusive e-cigarette use and dual use of cigarettes and e-cigarettes did not seem to be statistically significantly associated with SGA birth in our analysis, but this finding should be interpreted with caution given the low prevalence of use and the limited sample size. Considering the uncertainty of this nonsignificant association, future research using a larger sample size may be beneficial.

## Notes

Supplement 1.
eMethods.

eReferences.
**eFigure.** Sample Flowchart Among Pregnant Adolescents

Supplement 2.
Data Sharing Statement
