# Supplementary material for: Use of E-Cigarettes and Cigarettes During Late Pregnancy Among Adolescents
Source: JAMA Netw Open. 2023 Dec 13;6(12):e2347407. doi: 10.1001/jamanetworkopen.2023.47407 (PMC10719752; doi:10.1001/jamanetworkopen.2023.47407)
Supplement: Supplement 1. — eMethods. eReferences. eFigure. Sample Flowchart Among Pregnant Adolescents [file jamanetwopen-e2347407-s001.pdf]

Supplementary Online Content

Wen X, Liu L, Moe AA, et al. Use of e-cigarettes and cigarettes during late pregnancy among adolescents. *JAMA Netw Open.* 2023;6(12):e2347407. doi:10.1001/jamanetworkopen.2023.47407

eMethods.

eReferences.

eFigure. Sample Flowchart Among Pregnant Adolescents

This supplementary material has been provided by the authors to give readers additional information about their work.

© 2023 Wen X et al. *JAMA Network Open.*

eMethods.

Determinants of e-cigarette use

We chose these determinants as previous research showed that they were associated with cigarette use among pregnant adolescents[1,2] and/or e-cigarette use among pregnant young adults (aged 18-20)[3] or adults aged 18-49.[4] Note that we could not identify existing studies focusing on determinants of e-cigarette use among pregnant adolescents. Participants' education and household income were not included because most adolescents were not old enough to receive higher education and there was substantial missing data (23%) on household income.

Statistical analysis

In the descriptive analysis of the distribution of sociodemographic and pregnancy-related characteristics, we report frequencies and percentages for categorical variables as well as means and standard error (SE, weighted) for prepregnancy BMI (a continuous variable) overall and as a function of tobacco use group (exclusive cigarette, exclusive e-cigarette, dual cigarette/e-cigarette, none).

© 2023 Wen X et al. *JAMA Network Open.*

eReferences.

1. Cornelius MD, Leech SL, Goldschmidt L. Characteristics of persistent smoking among pregnant teenagers followed to young adulthood. *Nicotine Tob Res*. Feb 2004;6(1):159-69. doi:10.1080/14622200310001656975

2. Albrecht SA, Caruthers D. Characteristics of inner-city pregnant smoking teenagers. *J Obstet Gynecol Neonatal Nurs*. Jul-Aug 2002;31(4):462-9. doi:10.1111/j.1552-6909.2002.tb00069.x

3. Beck DC, Boyd CJ, Evans-Polce R, McCabe SE, Veliz PT. An examination of how e-cigarette/cigarette use during adolescence is associated with future use during the third trimester of pregnancy. *Subst Abus*. 2022;43(1):344-348. doi:10.1080/08897077.2021.1941519

4. Obisesan OH, Osei AD, Uddin SMI, et al. E-cigarette use patterns and high-risk behaviors in pregnancy: Behavioral Risk Factor Surveillance System, 2016-2018. *Am J Prev Med*. Aug 2020;59(2):187-195. doi:10.1016/j.amepre.2020.02.015

© 2023 Wen X et al. *JAMA Network Open.*

eFigure. Sample Flowchart Among Pregnant Adolescents

SGA - small-for-gestational-age.

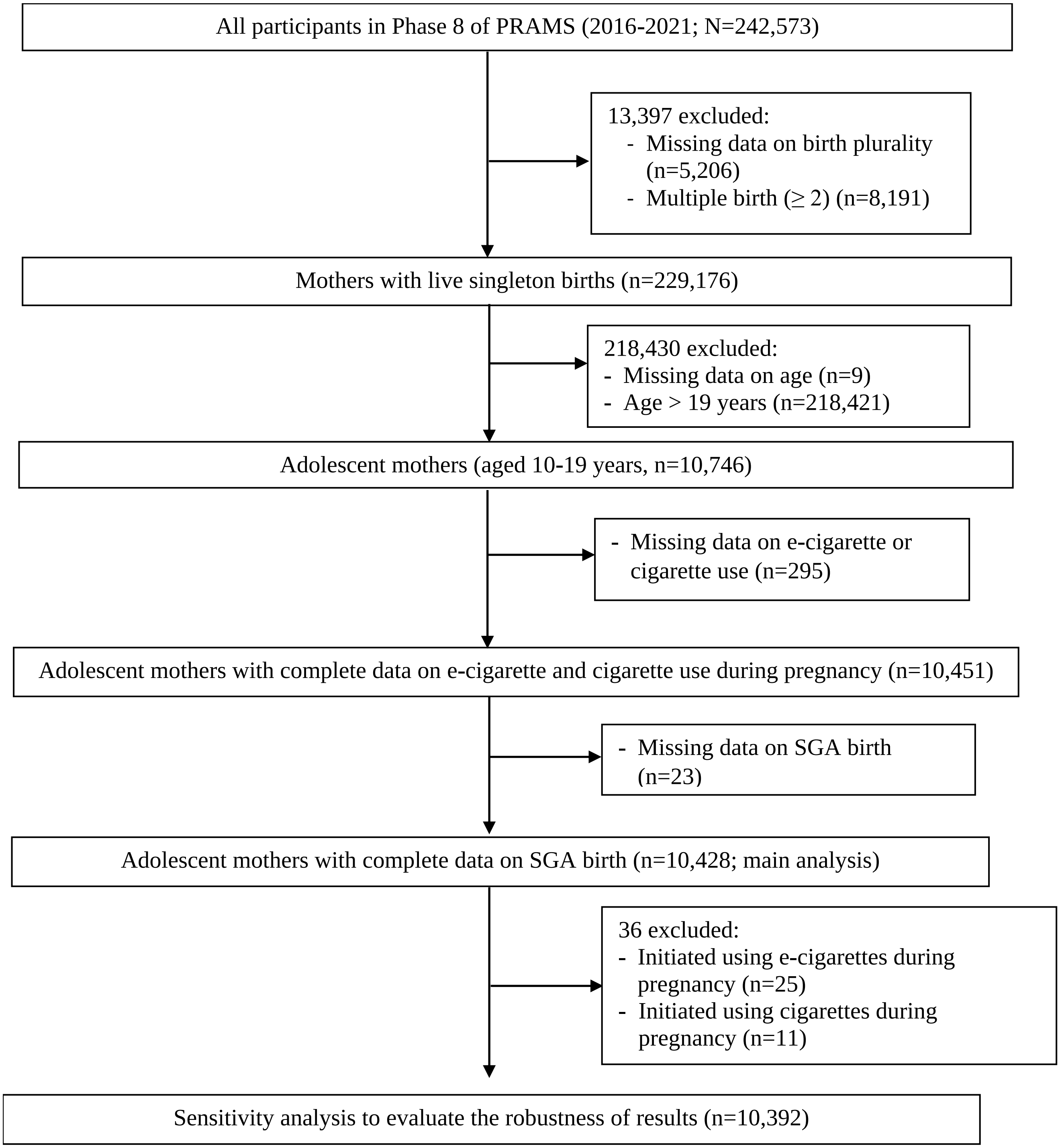

© 2023 Wen X et al. *JAMA Network Open.*
