# Supplementary material for: Use of E-Cigarettes and Cigarettes During Late Pregnancy Among Adolescents
Source: JAMA Netw Open. 2023 Dec 13;6(12):e2347407. doi: 10.1001/jamanetworkopen.2023.47407 (PMC10719752; doi:10.1001/jamanetworkopen.2023.47407)
Supplement: Supplement 2. — Data Sharing Statement [file jamanetwopen-e2347407-s002.pdf]

Data Sharing Statement

Wen. Use of E-Cigarettes and Cigarettes During Late Pregnancy Among Adolescents. *JAMA Netw Open*. Published December 13, 2023. doi:10.1001/jamanetworkopen.2023.47407

Data
Data available: No

Additional Information
Explanation for why data not available: The PRAMS data used for this study were provided by the Centers for Disease Control and Prevention (CDC). The authors are not allowed to share the data according to the signed External Researcher Data Sharing Agreement with the CDC.
